# Supplementary material for: Nurses and physicians attitudes towards factors related to hospitalized patient safety
Source: PLoS One. 2021 Dec 7;16(12):e0260926. doi: 10.1371/journal.pone.0260926 (PMC8651112; doi:10.1371/journal.pone.0260926)
Supplement: Attachment — Submitted filename: Response to Reviewers_PLOS ONE_27_09_2021-1.docx [file pone.0260926.s003.docx]

| Dear Reviewers,  The authors would like to thank the reviewer for his/her thorough review of the manuscript. We believe that this revised version, which includes reviewers’ suggestions, is more accurate and communicates better the main message of the article.  We will gratefully respond to any further comments on the text.  The paragraph is marked in yellow in the manuscript.  Thank you again for taking the time to review our paper and for your constructive comments.  Following comments have been modified in a new version of the manuscript:  Yours sincerely,  IML | | |
| --- | --- | --- |
| **Reviewer(s)' Comments to Author:** | | **Authors' response** |
| **Reviewer: 1** | I suggest that the authors make a correction in the last sentence of the second paragraph on page 3 which reads: “The analysis of factors contributing to the occurrence of adverse events is underlying condition for changes in the staff's behaviour, which may makes healthcare environment safer [7];” by replacing the word “makes” with “make” so that it will read as follows: “The analysis of factors contributing to the occurrence of adverse events is underlying condition for changes in the staff's behaviour, which may make healthcare environment safer [7]. | We would like to thank the Reviewer for his due remark. Has been changed. |
|  | In the “materials and methods” section in the fourth paragraph on page 5, I suggest that the authors delete the word “towards” in the following sentence: “Its first part includes questions related to the following subscales:: 1/ Teamwork climate -…….; 2/Safety climate - SC (questions from 7 to 13) – evaluates the perception of employees' organisational involvement in towards patient’s safety; 3/Job satisfaction - JS (questions from 15 to 19) –……”, so that it will read: “……..of employees' organisational involvement in patient’s safety;” | We would like to thank the Reviewer for his due remark. Has been changed. |
|  | In here, I suggest that the authors add a mention of also implementing the recently launched “Global Patient Safety Action Plan: 2021-2030”.  <https://wfsahq.org/news/latest-news/whas-global-patient-safety-plan-2021-30/> | We would like to thank the Reviewer for the suggestion to refer to the "Global Patient Safety Action Plan: 2021-2030" in this manuscript. Used in the text under “Discussion”, paragraph 9.  We have included the suggestion as follows:.  The recently introduced Global Patient Safety Action Plan: 2021-2030, which includes broadly developed strategies that can be adapted to the national context, can be used to do this. Their implementation will help ensure that patient safety is improved both nationally and globally. Therefore, all the more a safety culture should permeate the attitudes, beliefs, values, skills and practices of healthcare professionals, managers and leaders of healthcare organizations |
| **Reviewer: 2** | **Spelling, grammar and English need detailed review and edition.** | The text has been corrected. |
|  | **Title**  - Better if you specify specific factors  - It looks like attitude towards factors not patient safety  - It is very confusing title | We would like to thank the Reviewer for his attention. We changed the manuscript title to:  Nurses and physicians attitudes towards factors related to hospitalized patient safety |
|  | **Abstract**  - Key words seem like sentence | We would like to thank the Reviewer for his correct remark, the key words have been changed and clarified to:  safety, attitudes, patients, nurses, physicians |
|  | - Why medical and surgical nurses only | The research was carried out according to the protocol of the RN4CAST project, which included the study of a nurse from the surgical and medical departments. The study group worked in 21 hospitals representing a representative sample for Poland, and selected according to the geographical area of the country, population density, taking into account the hospital's reference level. The study was cross-sectional and correlational. In line with the assumptions of the RN4Cast project, internal medicine and surgery departments were selected because they provide multidisciplinary care provide health care to the majority of urgently hospitalized adult patients, provide multidisciplinary care and employ a large number of nurses. |
|  | **Introduction**  - Why you want to study this and how you do it differently are not stated well. | This research was conducted in accordance with the RN4CAST project protocol. Replication the RN4CAST protocol could help to analyse developing a culture safety. In addition, the inclusion of a new short safety assessment tool (SAQ questionnaire - the new tool used for this study) it is useful in the assessment of patient safety determinants by managers. When shaping a safe culture in health care organization, it should be remembered that ensuring the safety of patient care is primarily human resources - staff involved in the treatment process - mainly doctors and nurses. High awareness of occupational safety, cooperation in an interdisciplinary team, assessment of the culture of work safety drawing conclusions and making the right decisions may will increase the safety of hospitalized patients. On the other hand, it should be remembered that a strong safety culture is not only key to reducing harm to patients, but is also key to ensuring a safe working environment for healthcare professionals, which is why it is so important to undertake research to assess attitudes towards safety. |
|  | **Aim**  - What about other specific objectives | Specific objectives were included in the manuscript.  Detailed objectives:  1. What attitudes towards factors related to hospitalized patient safety are presented by nurses and by doctors?  2. What were the differences in attitudes towards safety in the group of nurses and doctors?  3. To what extent did the type of ward differentiate the attitudes towards safety of nurses and doctors?  4. What was the relationship between the respondents' gender and attitudes towards safety?  5. What was the relationship between the time of employment of the respondents and their attitudes towards safety? |
|  | **Methods and materials**  - Specify the validity and reliability of tool | As suggested by the Reviewer, the text of the manuscript has been supplemented in the "Material and methods" section, paragraph 3.  The Safety Attitudes Questionnaire reliability had a Cronbach's Alpha of 0.98. Before performing the analysis validity of the Polish adaptation of SAQ-SF, the Kaiser test was used to check whether the data meet the requirements of the factor analysis. The Kaiser-Mayer-Olkin (KMO) value, being the measure of the adequacy of the sample selection, was estimated at the level of 0.87 (df=8630, p<0.001). This model explained 68% of the total variance of the analysed set of variables (Malinowska – Lipień, et al. 2021). |
|  | What do you do to improve the quality of your tool? | The subsequent study is planned to improve the quality of the tool. |
|  | - How you select data collectors | At the hospital, a hospital coordinator was appointed to liaise with the research team and ensure consistency in data collection in line with the study guidelines. In clinical departments, the questionnaire was distributed among departmental staff by the hospital project coordinator. Participants had 4 weeks to complete the questionnaire. The completed anonymous questionnaires were deposited through an opening in securely closed boxes. Neither the coordinators nor anyone else at the hospital level had access to the contents of the boxes, they were only opened by a team of researchers. After 4 weeks, the coordinators collected the boxes, secured them and sent them by courier over to the research team. Before starting the study every participant was informed that participation was voluntary and anonymous, all responses are kept confidential and no individual responses would be available to the hospital management. |
|  | - You need comprehensive literature search to include all independent variables | The research results presented in the reviewed manuscript concern attitudes towards the patient safety of nurses and doctors assessed with the SAQ questionnaire. The independent variables were data obtained only from the SAQ questionnaire. The authors agree with the Reviewer that independent variables do not include all possible variables. There is no analysis regarding the age of the respondents. However, the questionnaire does not include such data.  The analysis was carried out in accordance with the principles described in the refences:  10. Bondevik GT, Hofoss D, Husebø SB, Tveter Deilkås EC. Patient safety culture in Norwegian nursinghomes. BMC Health Services Research. 2017, 17:424. doi 10.1186/s12913-017-23.  11. Milton J, Chaboyer W, Åberg ND, Andersson AE, Oxelmark L. Safety attitudes and working climate after organizational change in a major emergency department in Sweden. Int Emerg Nurs. 2020, 3;100830. doi: 10.1016/j.ienj.2020.100830.  12. Gabrani A, Hoxha A, Simaku A, Gabrani J. Application of the Safety Attitudes Questionnaire (SAQ) in Albanian hospitals: a cross-sectional study. BMJ Open. 2015;5:e006528, doi: 10.1136/bmjopen-2014-006528.  13. Jiang K, Tian L, Yan C, Li Y, Fang H, Peihang S, Li P, Jia H, Wang Y, Kang Z, Cui Y, Liu H, Zhao S, Anastasia G, Jiao M, Wu Q, Liu M. A cross-sectional survey on patient safety culture in secondary hospitals of Northeast China. PLoS One. 2019, 14(3):e0213055. doi:10.1371/journal.pone.0213055.  14. Bahar S, Önler E. Turkish surgical nurses' attitudes related to patient safety: A questionnaire study. Niger J Clin Pract. 2020, 23(4):470-475. doi: 10.4103/njcp.njcp_677_18. |
|  | - The operational definition is not clear, please write it clearly and in detail | As suggested, the definition according to WHO and The International Classification for Patient Safety (ICPS) was included.  For the WHO, “ patient safety means the reduction to an acceptable minimum level of risk of unnecessary harm related to health care”. The International Classification for Patient Safety (ICPS) defines ‘patient safety’ “as the act of avoiding, preventing or improving adverse outcomes or injuries occurred throughout the medical-hospital process” |
|  | - What about inclusion and exclusion criteria? | As suggested by the Reviewer, the text of the manuscript has been supplemented in the "Material and methods" section, paragraph 2.  The inclusion criterion was employment and active work during the study as a nurse or physician in a given hospital. All other hospital staff was excluded from the study.  Nurses on maternity leave, extended  sick leave or study leave were excluded from participation. |
|  | - How you minimize confounding factors | Potential confusing factors have been limited by through clear written instructions attached to the each questionnaire, and also by detailed familiarization of hospital coordinators with the meaning of the study and method of filling in forms, which was then handed over to participants. The research team provided necessary additional information and monitored the process of obtaining completed forms. |
|  | **Result, discussion and limitations**  - How you minimize correlation | Comparison of teamwork climate (TC), safety climate (SC), job satisfaction (JS), recognition of stress (SR), management perception (PM), working conditions (WC) between occupational groups, types of departments, genders, age groups patients covered by the care and seniority of nurses and doctors was first carried out using MANOVA - to take into account the influence of these factors on all aspects of safety attitudes at the same time, and taking into account the correlations between them. |
|  | - It’s difficult to add up the number of nurses and doctors’ response, how do you do it? | For some analyzes (e.g. unit type, gender, and work experience), the nurses 'and physicians' scores were summed up as both occupational groups completed the same SAQ. Both the questions of the questionnaire and the calculation method are the same for both occupational groups. |
|  | - How do you classify the age group? | In the studies, the study group was not divided by age, but only by work experience and the group of patients treated in the study unit (adults and adults & children). The time frame of work experience and the division into a group of patients are consistent with the original version of the SAQ. |
|  | - Why surgical and medical ward only | Studies for this manuscript were performed according to the RN4CAST project protocol. This study was carried out in a group of nurses working in departments, surgical and internal medicine departments, in 21 hospitals with 24-hour permanent duty, representative a representative sample for Poland, and selected according to the geographical area of the country, population density taking into account the hospital's reference level. The study was cross-sectional and correlational. In line with the RN4Cast project, the medical and surgical departments were selected because they provide multidisciplinary care and employ a large number of nurses. |
|  | - You need to explain the possible reasons in study differences and similarities | Included in the manuscript.  Polish nurses received higher mean score in the teamwork subscale ~~mean results in the subscale concerning teamwork~~ (TC) (62.38 pts.) than nurses working in Albania (45.7 pts.) [12] and Turkey (46.27 pkt.) [14], but critically lower compared to European countries as Norway and Sweden [10-11], Asia (China, Saudi Arabia, Iran) [13, 15-16], Americas (Brazil, and US-PA, Pittsburgh) [18-19], Australia [9] or Africa (Kenya) [17]. The evaluation of team work by Polish nurses may result from the fact that position of physician is being prioritised in the hierarchy of the Polish healthcare system. This opinion has an impact on the teamwork and may prevent equal contribution of skills and knowledge of ~~from other health team members those~~ team members who are not doctors. Effective cooperation of various professionals in a team constitutes an important element of care and their patient safety.  In the aassessment of safety climate (SC), Polish nurses received higher mean results (63.41 pts.) than nurses working in Albania (36.8 pts.) [12] and Turkey (46.44 pkt.) [14], but definitely lower mean values than nurses working in Norway or Sweden [10-11], as well as in the area of Asia (China, Saudi Arabia, Iran) [13, 15-16], Americas (Brazil, and the state of Pennsylvania US-Pittsburgh) [18-19], Australia (Australia) [9] or Africa (Kenya) [17]. Within the safety climate (SC) subscale, Polish physicians obtained higher mean results compared with the physicians ~~doctors~~ from Sweden and Albania [11-12] |
|  | - Some of works you should do did not need to be stated as limitations | The presented limitations of the work result from other assumptions of the presented research and the lack of available data. The authors consider these issues to be important to consider in future research (e.g., different types of wards, more hospitals, different medical professions). The authors believed that these were more limitations rather than the results of the research performed. Limitations, the reduction of which in subsequent studies will allow for a better understanding of the factors that determine the patient's safety. |
