# Supplementary material for: Nurses and physicians attitudes towards factors related to hospitalized patient safety
Source: PLoS One. 2021 Dec 7;16(12):e0260926. doi: 10.1371/journal.pone.0260926 (PMC8651112; doi:10.1371/journal.pone.0260926)
Supplement: Attachment — Submitted filename: plos review.docx [file pone.0260926.s004.docx]

- Thank you for addressing most of the comments
- Still your English and grammar need detailed review and edition

**Title**

- Why nurses and doctors were your priority? What about other health professionals?
- “Nurses and physicians’ attitudes towards factors related to hospitalized patient safety”, please specify what factor you intend to study?
- “What about other staff nurses…” need further explanation?

**Methods**

- Operationalize patient safety, attitude in quantitative way (quantify it)
- How you minimize confounding factors needs further explanation
- Which age group is adult and which one is children
