# Supplementary material for: Nurses and physicians attitudes towards factors related to hospitalized patient safety
Source: PLoS One. 2021 Dec 7;16(12):e0260926. doi: 10.1371/journal.pone.0260926 (PMC8651112; doi:10.1371/journal.pone.0260926)
Supplement: Attachment — Submitted filename: Response to Reviewers_31-10-2021.docx [file pone.0260926.s005.docx]

*Author Response*

*The authors would like to thank the reviewer for his/her thorough review of the manuscript. We believe that this revised version, which includes reviewers’ suggestions, is more accurate and communicates better the main message of the article.*

*We will gratefully respond to any further comments on the text.*

*The paragraph is marked in green (English and grammar) and yellow (other changes) o in the manuscript.*

*Thank you again for taking the time to review our paper and for your constructive comments.*

*Best regards,*

**Reviewer #1:**

One typing error for correction in the fourth (04th) paragraph of “Discussion section” - the first sentence, which reads: “In the aassessment of safety climate (SC), Polish nurses received…….”. Spellings for the word assessment need to be corrected.

The text was checked in terms of language and grammar. The corrected text is highlighted in green in the manuscript.

**Reviewer #2:**

- Still your English and grammar need detailed review and edition

The text was checked in terms of language and grammar. The corrected text is highlighted in green in the manuscript.

**Title**

- Why nurses and doctors were your priority? What about other health professionals?

In Poland, the largest group of medical professions are nurses and doctors. It is doctors and nurses who play the main role in the process of diagnosis and treatment and patient care. According to the report of the National Health Fund, he works in hospitals (<https://ezdrowie.gov.pl/portal/home/zdrowe-dane/zecja/zecja-dotyczace-liczby-personelu-medycznego-w-umowach-z-narodowym-funduszem-zdrowia> ):

**nurses - 192 223**

**doctors - 113 724**

physiotherapists - 38 416

midwives - 25 033

laboratory diagnosticians - 760

pharmacists - 18 564

In the "Research limitations" part, the question of extending the study group to include representatives of other medical professions was taken into account. Therefore: "In addition, in subsequent studies, to accurately assess the accuracy of the scale, it should also be tested by comparing different groups of medical professions, and their selection would take into account a comparable number of respondents."

- “Nurses and physicians’ attitudes towards factors related to hospitalized patient safety”, please specify what factor you intend to study?

The research used the international SAQ scale, which allows the assessment of patient safety in 6 areas: 1/Teamwork Climate; 2/ Safety Climate; 3/ Job Satisfaction; 4/Stress Recognition; 5/Perceptions of Management; 6/ Working Conditions. The research was carried out on the basis of the SAQ questionnaire adapted to Polish conditions (Malinowska-Lipień I, Brzyski P, Gabryś T, Gniadek A, Kózka M, Kawalec P, et al. Cultural adaptation of the Safety Attitudes Questionnaire - Short Form (SAQ-SF) in Poland. PLoS One. 2021 Feb 5;16(2):e0246340. doi: 10.1371/journal.pone.0246340).

- “What about other staff nurses…” need further explanation?

In Poland, according to the report of the Ministry of Health on a national scale, the largest number of beds was intended for patients requiring surgical treatment - 39.3 thousand. and for internal medicine patients - 23.0 thousand (Report - pp. 44 - 45, chart 18 (English version), therefore it was decided that the research should focus on this group of nurses and doctors. Of course, it is a correct remark that in subsequent studies it is necessary to include staff working in all types of wards, this was taken into account parts in "Research limitations" (Report: <https://stat.gov.pl/files/gfx/portalinformacyjny/pl/defaultaktualnosci/5513/1/9/1/zdrowie_i_ochrona_zdrowia_w_2018.pdf>)

“Subsequent future studies should expand research to a larger number of hospitals and different types of wards (including pediatric, intensive care and psychiatric) with additional inclusion of the influence of factors conditioning attitudes towards safety on the frequency of adverse events, including international studies comparing different countries”.

**Methods**

- Operationalize patient safety, attitude in quantitative way (quantify it)

The test results were presented both as quantitative (Table 1) and qualitative (Table 2) data. The results of own research and quantitative data were compared in the discussion with the results of research carried out in 11 different countries. In all these studies, quantitative data were presented, our results were additionally enriched with qualitative data presented in Table 3. The results of the individual 6 subscales were divided into> = 75 points and <75 points, where * ≥75 pts. - positive result. <75 pts.- negative result.

- How you minimize confounding factors needs further explanation

The "Materials and methods" section describes the data collection process in detail, ensuring such conditions minimized disruptive factors.

At the hospital, a hospital coordinator has been appointed to work with the research team and ensure consistency in data collection in line with the guidelines of the study. In clinical departments, the questionnaire with a plastic envelope attached to it was distributed during departmental staff meetings led by the hospital project coordinator. Participants had 4 weeks to complete the questionnaire. Completed questionnaires packed in a sealed plastic envelope were thrown into a sealed box with a hole. After 4 weeks, the coordinator was responsible for collecting the boxes, securing them and handing them over to the research team. Participants were informed that participation was voluntary and anonymous, that all responses would be kept confidential and that no individual responses would be available to hospital management.

- Which age group is adult and which one is children

The SAQ questionnaire in the "BACKGROUND INFORMATION" section has a question about the majority of patients in the ward. There are three distractors 1- adults; 2 kids; 3- both groups (original version of the SAQ: <https://med.uth.edu/chqs/wp-content/uploads/sites/75/2020/03/SAQ-Short-Form-2006.pdf> ; Polish version of SAQ: <https://journals.plos.org/plosone/article?id=10.1371/journal.pone.0246340> ). In Poland, adult departments cover patients from 18 years of age, and children's departments up to 18 years of age.
