# Supplementary material for: Nurses and physicians attitudes towards factors related to hospitalized patient safety
Source: PLoS One. 2021 Dec 7;16(12):e0260926. doi: 10.1371/journal.pone.0260926 (PMC8651112; doi:10.1371/journal.pone.0260926)
Supplement: Attachment — Submitted filename: Response to Reviewer_16-11-2021.docx [file pone.0260926.s006.docx]

Krakow, 16.11.2021

Dear Reviewer,

The authors would like to thank the reviewer for review of the manuscript.

We believe that this revised version, which includes reviewers’ suggestions, is more accurate and communicates better the main message of the article.

We will gratefully respond to any further comments on the text.

The article has been linguistically proofread by a professional proof reader. In the manuscript, the corrected text is highlighted in yellow.

Thank you again for taking the time to review our paper and for your constructive comments.

Best regards,

Iwona Malinowska-Lipień
